# Effect of meniscus modelling assumptions in a static tibiofemoral finite element model: importance of geometry over material

**DOI:** 10.1007/s10237-024-01822-w

**Published:** 2024-02-13

**Authors:** Jiacheng Yao, John Crockett, Mathias D’Souza, Gavin A. Day, Ruth K. Wilcox, Alison C. Jones, Marlène Mengoni

**Affiliations:** Institute of Medical and Biological Engineering, School of Mechanical Engineering, University of Leeds, Leeds, UK

**Keywords:** Tibiofemoral joint, Meniscus, Sensitivity, Contact mechanics

## Abstract

Finite element studies of the tibiofemoral joint have increased use in research, with attention often placed on the material models. Few studies assess the effect of meniscus modelling assumptions in image-based models on contact mechanics outcomes. This work aimed to assess the effect of modelling assumptions of the meniscus on knee contact mechanics and meniscus kinematics. A sensitivity analysis was performed using three specimen-specific tibiofemoral models and one generic knee model. The assumptions in representing the meniscus attachment on the tibia (shape of the roots and position of the attachment), the material properties of the meniscus, the shape of the meniscus and the alignment of the joint were evaluated, creating 40 model instances. The values of material parameters for the meniscus and the position of the root attachment had a small influence on the total contact area but not on the meniscus displacement or the force balance between condyles. Using 3D shapes to represent the roots instead of springs had a large influence in meniscus displacement but not in knee contact area. Changes in meniscus shape and in knee alignment had a significantly larger influence on all outcomes of interest, with differences two to six times larger than those due to material properties. The sensitivity study demonstrated the importance of meniscus shape and knee alignment on meniscus kinematics and knee contact mechanics, both being more important than the material properties or the position of the roots. It also showed that differences between knees were large, suggesting that clinical interpretations of modelling studies using single geometries should be avoided.

## Introduction

The use of computational models in healthcare is increasing, for a range of purposes including research and development, preclinical testing and clinical use. In any of these applications, there is a need to understand the context in which specific computational models have been developed, their applicability and limitations (Mengoni 2021; Viceconti et al. 2021). For example, in the case of interventions for knee osteoarthritis, finite element models of the knee may be developed to assess specifically the effect of the intervention on knee kinematics (e.g. Mootanah et al. 2014; Steineman et al. 2020; Shriram et al. 2021), or on cartilage pressure (e.g. D'Lima et al. 2009; Khoshgoftar et al. 2015; Xu et al. 2022) but may not be accurate for both types of outputs. Further, while validation studies often replicate an equivalent experimental (or computational) protocol, they often do not report on how sensitive the model outputs are to its inputs or which assumptions can be modified and still produce a “valid” outcome. Understanding the sensitivity of results to input parameters can contribute to defining the contexts for which a modelling methodology remains valid, and those for which it should not be used.

For example, for tibiofemoral models developed to analyse the stress or contact distribution in the knee cartilage, there is often little information provided on the effect of the assumptions made to model the menisci. In this type of model, the meniscus is often modelled as a transversely isotropic linearly elastic material (as reviewed in Imeni et al. 2020), with the menisci circumferential modulus three to ten times larger than the axial modulus, and little variation in the latter (e.g. Meakin et al. 2013; Zielinksa and Haut Donahue 2006; Carey et al. 2014; Klets et al. 2016; Meng et al. 2017; Steineman et al. 2020 all use a 20 MPa axial modulus, with a circumferential modulus varying between 120 and 200 MPa). While there has been analysis of the effect of this variation on the outcomes for the meniscus (Steineman et al. 2020), little has been reported on the assessment of the effect of meniscus modelling choices on the joint contact behaviour. Without this analysis, it is difficult to compare different studies to identify sources of variation in contact mechanics.

The aim of this work was to assess the sensitivity to geometrical and material assumptions used to model the meniscus for finite element models of the tibiofemoral joint developed for contact mechanics.

## Methods

A generic model of a tibiofemoral condyle was used to assess the effect of the meniscus attachment shape on the contact mechanics and meniscus kinematics. In parallel, specimen-specific finite element models of human tibiofemoral joints, valid for cartilage contact mechanics, were used in a one-at-a-time sensitivity analysis to understand the effect of joint alignment and meniscus shape, material model parameters and attachment properties. All finite element models were nonlinear quasi-static and run with Abaqus 2019 (Simulia, Dassault Systèmes).

### Generic models

Using the first generation Open-Knee model (Erdemir 2013), methods were developed to alter the type of meniscus root attachment of a single medial condyle model containing cartilage layers and meniscus only (Meng et al. 2017). The cartilage was modelled as a neo-Hookean solid (Cooper et al. 2020 and Cooper et al. 2023). The meniscus was modelled as a transversely isotropic linear elastic material (Table 1, baseline values) with an axial modulus equal to the equivalent elastic modulus of the cartilage and using a ratio between the circumferential and axial moduli of 3.5 (Cooper et al. 2023). A load of 250 N was applied to the proximal surface of the femoral cartilage alongside the femoral axis with other degrees of freedom fixed. The distal surface of the tibial cartilage was completely fixed. The cartilage and meniscus were meshed with linear hexahedral elements of reduced integration, with an element size of approximately 0.7 mm (Meng et al. 2017).

**Table 1:** Elastic modulus values of a transverse isotropic material model for the meniscus when the equivalent cartilage elastic modulus is 6 MPa—sensitivity of transverse and circumferential ratios, where R is the ratio of circumferential to transverse meniscus moduli, and M is the ratio of transverse meniscus to cartilage moduli

Model label	Baseline	A	B	C	D	E
Elastic modulus in the transverse direction (MPa)	6	12	18	6	6	6
Elastic modulus in the circumferential direction (MPa)	21	42	63	42	48	60
	M = 1	M = 2	M = 3	R = 7	R = 8	R = 10
	R = 3.5			M = 1		

Four root configurations were developed: (G1) Using linear springs connecting each node of the truncated end of the meniscus to a single point on the tibial plateau, with the total spring stiffness equivalent to a material elasticity equating that of the meniscus in the circumferential direction (baseline model); (G2) replacing the linear springs by rigid connectors (kinematic coupling between the cartilage and the truncated end of the meniscus); and (G3 and G4) modelling explicit 3D shapes to anchor the truncated meniscus end surfaces to the tibial plateau: The 3D shapes were obtained by lofting from the truncated meniscus extremity to a circular area, along a path following the curvature of the medial cartilage. The root circular attachment surface areas were defined depending on root location representing the range of core root areas measurements in the literature (Ellman et al. 2014), creating (G3) small and (G4) large attachment areas (Table 2). This did not necessarily create a realistic root shape, but the method was used to assess the importance of the shape of the root with respect to the simplified models. The 3D root material was modelled as linearly elastic with a Young’s modulus of 21 MPa and Poisson’s ratio of 0.49. The roots were meshed with tetrahedral quadratic elements with an element size of approximately 0.8 mm, with a mesh convergence study yielding displacements and contact pressure changes lower than 1% when halving the size of elements.

**Table 2:** Core attachment surface areas used in models G3 (small areas) and G4 (large areas) (Ellman et al. 2014)

	Small area (mm^2^)	Large area (mm^2^)
Anterior root	45.0	70.0
Posterior root	32.8	51.1

### Specimen-specific models

**Fig. 1:**
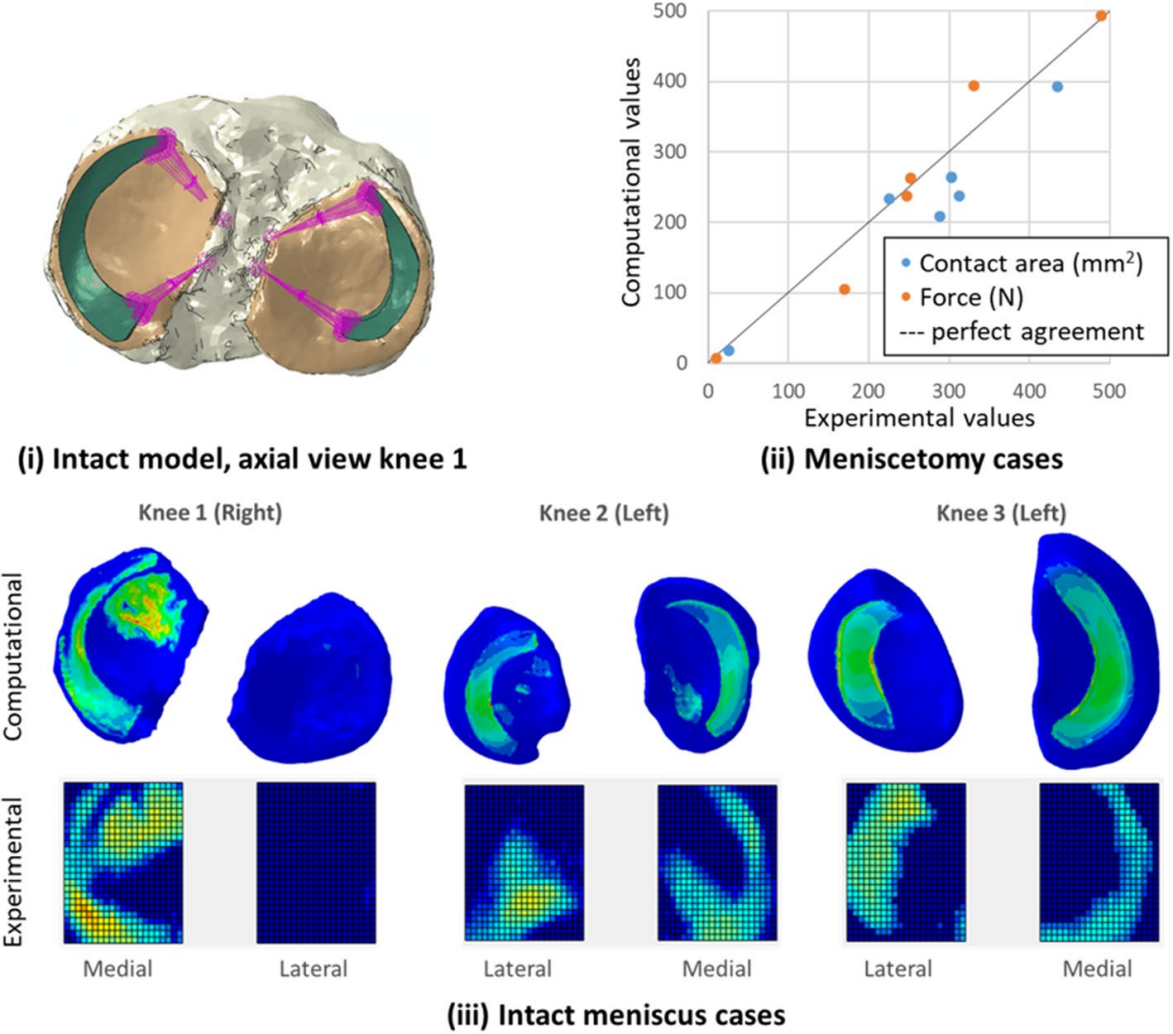
Baseline data for this sensitivity study (Cooper et al. 2023). **i** Example axial view of an intact baseline model with the menisci (green) and their spring attachments (purple) to the tibial plateau (beige); **ii** comparison of experimental and computational values for two outputs of interest (contact area and contact force) on each condyle for double meniscectomy cases; **iii** qualitative comparison of computational and experimental contact areas for each knee

In parallel, specimen-specific models of three human tibiofemoral joints were derived from previous work (Cooper et al. 2023) where they had been developed and the contact mechanics compared to in vitro data of the same knees in axial compression and full extension. Briefly, the models were based on MRI and CT data of the corresponding experimental specimens. The baseline meniscus model had a geometry based on MR and CT images, truncated to represent the meniscus roots each modelled with 15 linear springs attached to one point on the tibia (Fig. 1i). The meniscus and cartilage material model for the specimen-specific baseline FE models were modelled in the same way as for the generic baseline model (Table 1). The bones were modelled as rigid solids. The tibia was completely fixed, and a load of 500 N was applied alongside the femoral axis with all other degrees of freedom completely fixed.

In this previous study (Cooper et al. 2023), the contact force through each condyle and the total contact area on the tibial plateau for each condyle (contact between meniscus and tibial cartilage and between femoral cartilage and tibial cartilage) were evaluated for knees tested without menisci and with the menisci retained and compared to specimen-specific experimental data. Differences were within experimental error on most outputs for the double meniscectomy cases (Fig. 1ii). For the intact cases, the predicted contact pressure distributions were qualitatively well matched to the experimentally measured values (Fig. 1iii); quantitative comparison was not possible due to the contact extending beyond the edges of the pressure sensor experimentally.

In this study, a one-at-a-time sensitivity analysis was performed on each of the three knees (Table 3) for (S1) the values of the axial or circumferential moduli of the meniscus (five variations for each knee), for (S2) the root attachment location and shape (four variations), for (S3) the shape of the meniscus (1 variation) and for (S4) the relative alignment of the femur to the tibia (1 variation).

1. Effect of material properties

**Table 3:** Summary of variables of interest in the 4 sensitivity studies (S1 to S4) for the three image-specific knees (leading to three times 11 models besides the three baseline models)

	Variable of interest	Baseline value	Value	Labels
S1	Ratio of meniscus axial modulus to cartilage modulus	1 (“M” in Table 1)	2	A
			3	B
	Ratio of circumferential to axial elastic moduli in the meniscus	3.5 (“R” in Table 1)	7	C
			8	D
			10	E
S2	Spring attachment position on the tibia	Position so that average spring length is 10 mm	Anatomical	F
			Translated by 1 mm from F	G
	Spring attachment shape on the tibia (15 springs per root)	Single point attachment	Along core circumference	H
			Along total circumference	J
S3	Meniscus segmentation	Simplified from MRI	MRI accurate	K
S4	Joint orientation	Full extension	20° flexion from K	L

The range of material parameters tested (Table 1) was based on variation of values in the literature (Cooper et al. 2020; Meakin et al. 2013; Zielinksa and Haut Donahue 2006; Carey et al. 2014; Klets et al. 2016; Meng et al. 2017; Steineman et al. 2020), creating models where either the transverse modulus of the meniscus was changed with respect to the equivalent modulus of the cartilage (models labelled A and B), or the circumferential modulus of the meniscus with respect to its transverse modulus (models labelled C to E). Shear moduli values that depend on the elastic moduli were adjusted accordingly. The spring stiffness values for the root attachments were modified when the circumferential modulus of the meniscus was changed, so that the total stiffness of the root represented a material with a modulus equivalent to the meniscus circumferential modulus.

(S2) Effect of root attachments

The root attachment position in the baseline model was defined based on a prescribed length of the springs attaching each meniscus root to one point on the tibial plateau. Four other configurations were considered in this sensitivity study (Table 4): (models labelled F) A single point attachment on the tibial plateau at an anatomical position defined at a generic distance from the centre of the tibial tuberosity and the lateral or medial tibial eminence (LaPrade et al. 2014 and Johannsen et al. 2012); (models labelled G) a single point attachment slightly away from the anatomical location, representative of what could happen in a meniscus graft surgery; (models labelled H and J) a surface attachment where the springs were attached alongside a circumference encompassing an average core or total root attachment area (LaPrade et al. 2014 and Cruz et al. 2017).

**Table 4:** Core and total root attachment area centred around anatomical positions (TT, tibial tuberosity; LTE lateral tibial eminence and MTE, medial tibial eminence) (LaPrade et al. 2014 and Cruz et al. 2017)

Root	Core attachment area (mm^2^)	Total attachment area (mm^2^)	Anatomical position
Posterior			
Medial	30	78	9.6 mm posterior of MTE
			0.7 mm lateral of the MTE
Lateral	39	115	1.5 mm posterior of LTE
			4.2 mm medial of the LTE
Anterior			
Medial	N/A	141	27.5 mm anterior of MTE
			27 mm medial to TT
Lateral	56	110	18 mm anterior to MTE
			14.4 mm lateral to LTE

(S3) Effect of meniscus shape

**Fig. 2:**
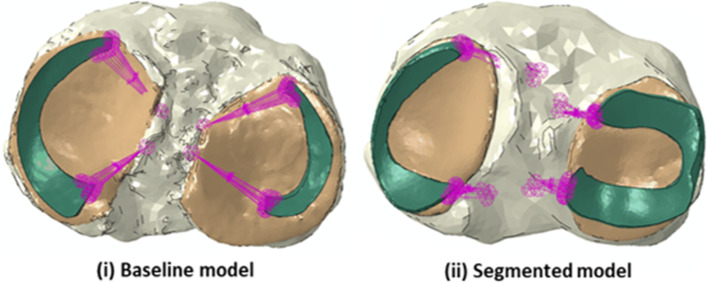
Comparison of meniscus shapes and corresponding attachments: **i** baseline model and **ii** “segmented” model of the same knee (models K)

In the baseline model (Cooper et al. 2023), the meniscus shape had been simplified to ensure convergence of FE model solutions in free rotations conditions (not used here). In this study, it was possible to use a more realistic shape of the meniscus (Fig. 2), creating a model referred to as “segmented” (models labelled K). The meniscus was segmented from MRI DESS images (3T Siemens Magnetom Prisma, Erlangen, Germany with a 3D Double Echo Steady State sequence, at a resolution of 0.36 × 0.36 × 0.7 mm^3^), with adjustments on the axial direction to maintain a surface conforming to the cartilage surface. The springs representing the roots were attached to the anatomical position defined in S2.

(S4) Effect of knee alignment

Finally, the full extension of the knee which had been used to replicate the in vitro testing conditions were relaxed to a mid-stance 20° angle between the tibial axis and the femoral axis (models labelled L). This change of alignment was done with respect to the “segmented” models with additional adjustments in the segmentation of the meniscus to maintain conformity with cartilage surfaces after rotation.

### Outputs of interest

Finite element outputs analysed were those used in the baseline study (Cooper et al. 2023), total contact area on each condyle (two values per model) and contact force ratio through each condyle (two values per model), with the addition of the maximum relative displacement of the meniscus with respect to the cartilage on each tibial plateau (one value per model) and, where relevant, the maximum stretch values of the linear springs across the 60 springs (one value per model).

To compare the effect of the four different aspects considered in the sensitivity study (material parameters, root attachment representation, segmentation and orientation), a Kruskal–Wallis test was performed for the contact area (only output of interest with N > 3 in all four groups), with post hoc Mann–Whitney tests and Bonferroni corrections for significance level of 0.01, using R version 4.2.2.

## Results

### Generic models

The baseline model (with roots assumed to behave as springs) led to a contact area of 491 mm^2^ and a maximum relative displacement of the meniscus of 2.15 mm.

**Fig. 3:**
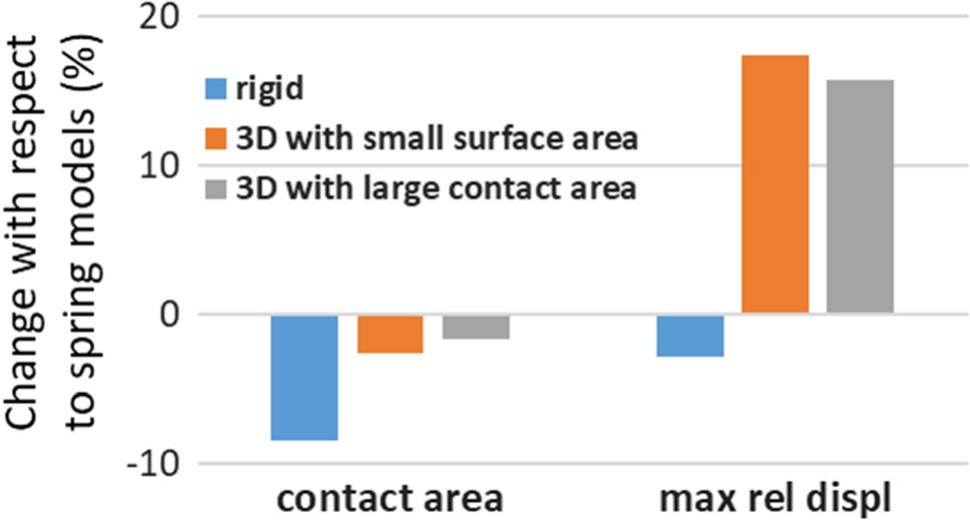
Generic model—effect of meniscus attachment modelling assumption on a single condyle contact area and meniscus displacement. Percentage changes with respect to a model where the meniscus is attached with a series of spring

Modelling the root attachments with a rigid connector reduced the contact area by over 8% with respect to that of the baseline model, whereas modelling the roots as a 3D structure yielded differences in contact area smaller than 3% (Fig. 3). The 3D structures had a significant effect on the relative motion of the meniscus (Fig. 3) with an increase of over 15% from the baseline.

### Specimen-specific models

**Fig. 4:**
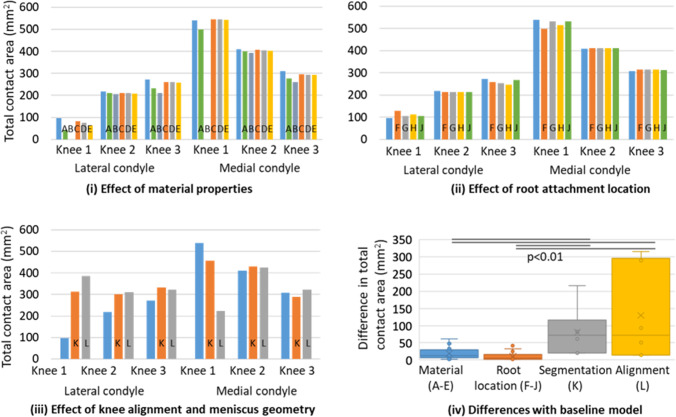
Specimen-specific models—effect of modelling assumptions on the total contact area in each condyle. For each of the condyle, the first data point (in blue) is for the baseline model and data is labelled A to L as per methods. **i** Outcomes of the five additional material configurations. Note that the first knee did not provide contact on both condyles in model B; **ii** outcomes for the four additional root attachment locations; **iii** outcomes for the change in meniscus segmentation (orange) and in added joint alignment (grey); **iv** comparison of the difference between models and the baselines for all models related to the change in material properties, root attachment location, segmentation of the meniscus and knee alignment, with information on statistical significance after Bonferroni corrections

The total contact area for the three specimen-specific models showed little sensitivity to the material properties of the menisci (Fig. 4i) or to the location of their root attachment (Fig. 4ii), with a smaller effect due to these changes than due to the differences between knees. Of these, the sensitivity to the axial modulus values showed the most systematic effect, with a decrease of the contact area when the axial modulus of the meniscus increased (data points D and E on each condyle in Fig. 4i). The total contact area for each condyle was more affected by adjusting the menisci segmentation (models K), with an added effect of adjusting the joint alignment from a full extension to a mid-stance position (models L) (Fig. 4iii). These differences were prominent for the first knee which had a small baseline contact area on the lateral condyle. The effect of adjusting the segmentation or alignment was significantly larger than the effects caused by other changes (Fig. 4iv), with no significant difference between a change of segmentation only and the additional change in alignment (alpha values of Mann–Whitney post hoc tests: Materials v root location 0.0128; material v segmentation 0.0027; material v alignment 0.0023; root location v segmentation 0.001; root location v alignment 0.0021; segmentation v alignment 0.9362).

The sensitivity on the force distribution between condyles (Table 5) followed a similar pattern to that of the total contact area on each condyle. The material parameter changes and the root location changes led to differences in contact forces much smaller than those produced by the changes due to the segmentation or the alignment.

**Table 5:** Ratio of force through the medial condyle across three knees and 12 configurations

	Baseline value [%]	Material parameters (A–E) [%]	Root location (F–J) [%]	Segmentation (K) [%]	Alignment (L) [%]
Knee 1	98.6	99.5–99.8	98.2–98.3	63.4	40.2
Knee 2	65.0	64.8–65.6	64.2–64.5	67.8	59.7
Knee 3	53.8	51.9–56.1	54.9–56.5	53.4	59.4

**Fig. 5:**
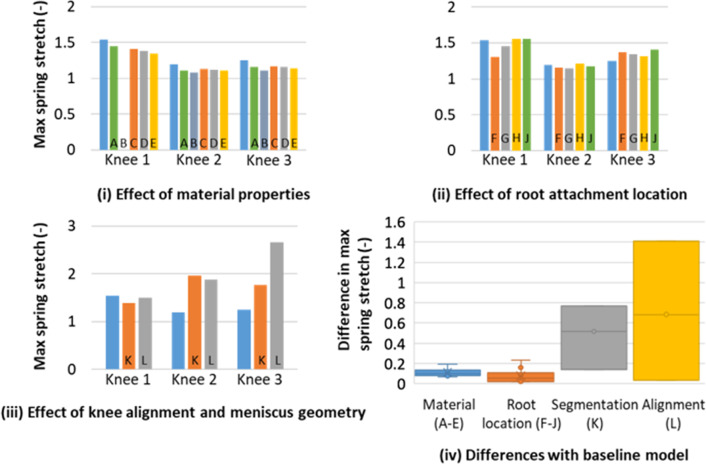
Specimen-specific models—effect of modelling assumptions on the root spring stretch. Data visualisation as per Fig. 4

**Fig. 6:**
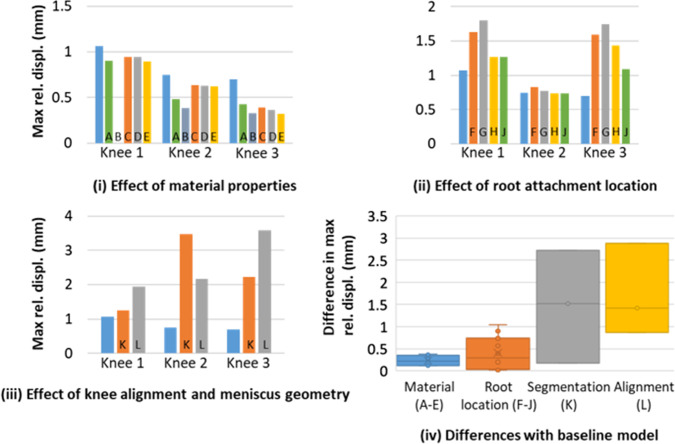
Specimen-specific models—effect of modelling assumptions on the relative displacement of the menisci on the tibia. Data visualisation as per Fig. 4

The springs’ stretch and maximum displacement of the menisci on the tibia were more sensitive to the material properties (Figs. 5i and 6i) or to the location of the root attachment (Figs. 5ii and 6ii) than the contact outputs were, with the sensitivity of the segmentation (Figs. 5iii and 6iii) and alignment (Figs. 5iv and 6iv) still dominating the effects.

## Discussion

In this work, a systematic sensitivity analysis was performed to assess the effect of meniscus modelling assumptions on the contact behaviour of tibiofemoral finite element models, as well as on the displacement of the meniscus in such models. It demonstrated the importance of the meniscus shape over that of material parameters.

The contact mechanics were quantified here by the contact area on each condyle and the force ratio through each condyle. With both these measures, variations in the meniscus segmentation and in the joint alignment had effects two to six times larger than variations in the material parameters used for the meniscus or in the assumptions made for root attachments. When assessed on specimen-specific models, the differences due to variations in the meniscus segmentation and in the joint alignment were also larger than the differences in outcomes seen between knees.

The local meniscus movements were quantified here as the maximum relative displacement between menisci and the tibia and by the maximum stretch in the menisci root attachments. With these measures, the different variations tested in this work did not have significant differences in outcomes. However, the variance between knees increased with a more precise segmentation of the meniscus, suggesting it could have a large effect on meniscus displacement. Moreover, when assessed on a generic single-condyle model, the shape of the root appears to be important for the observed meniscal displacement.

While modelling assumptions for the meniscus roots have been shown to affect the contact distribution (Haut Donahue et al. 2003), they have less influence on the peak contact pressure (Rooks et al. 2022) which is driven by the knee geometry and FE mesh. The current work shows that these assumptions have also little effect on the total contact area, when the joint is constrained. A previous sensitivity study on a single knee also showed that the material properties of the meniscus were less important than their attachment in modelling meniscus displacement (Yao et al. 2006). One sensitivity study analysed the effect of using linear versus non-linear isotropic material models for the meniscus on the tibial cartilage contact pressure (Elmukashfi et al. 2022). It showed, using one knee model, that the contact pressure difference was small, with similar distribution but higher peak contact pressure and smaller contact area for the linear models, and that the majority of these changes occurred at the meniscus attachment sites. Studies that have shown that some material parameters were more important than others for the contact mechanics (e.g. Haut Donahue et al. 2003 and Yao et al. 2006), did not directly compare this to the importance of the shape or orientation.

While this sensitivity study was conducted on a limited number of anatomical variations (one generic model and three specimen-specific models), clear trends in the effect of meniscus modelling assumptions were observed. Within the tested variations of material parameters and root configurations, the changes for all outputs of interest were below or within the observed variation seen between knees using the baseline models. Conversely, the tested variations for the meniscus shape and the knee alignment resulted in differences that were larger than the differences between individual knees.

With all models being highly constrained (only degree of freedom being in the direction of the applied force), large differences in behaviour were seen between knees in the model outcomes. This was true both for the baseline model and the variations in the sensitivity studies. In particular, knee 1 was loaded in such a way that the baseline model (and corresponding experiments in the previous work (Cooper et al. 2023)) had most of the load going through only the medial condyle, while the other two knees had a more balanced distribution of loads. This led to a much larger variation in behaviour for knee 1 when adjustments were made to the meniscus segmentation or the joint orientation, compared to the changes observed in the other two knees.

Few studies provide direct comparison of contact behaviour between knees, for which samples have been tested experimentally with the same methodology or have been modelled computationally with a consistent methodology. The variation observed between knees in this study mirrors the qualitative differences in experimental contact distribution noted across three knees tested at varying flexion angles (Beidokhti et al. 2017), for which some condyles did not experience contact at all. The variation in computational contact area in this work is smaller than that measured experimentally across seven knees tested at their most natural alignment (Fukubayashi and Kurosawa 1980). The variation observed between two knees with similar constraints to the present study (Gu and Pandy 2020) was less pronounced than that seen in this work.

The variation in behaviour between knees, and dependence on specific joint shape and orientation, highlights that care should be taken when comparing modelling outcomes with previous published studies as a validation or verification process. As such, a “valid” comparison of modelling outcomes with single values from the literature is likely to be related to coincidence in reproducing with one shape what was obtained with another shape. However, a comparison with a range of values from the literature is likely to create an artificially large target for model validation. The confidence in validation processes for image-based models would be increased by comparing modelling outcomes directly with experimental outcomes of the same joints. Similarly, interpreting results of studies with a single image-based model should be done cautiously as the mechanical role of the meniscus may be quite different from one knee to another. In particular, clinical interpretation of modelling studies using single geometries should be avoided.

The findings of this sensitivity study have significant consequences when developing models through an inverse modelling analysis used for calibration of material parameters (e.g. De Rosa et al. 2022; Elmukashfi et al. 2022; Long et al. 2022). Unless specimen-specific knee models can be developed and compared to their corresponding experimental data, the calibration of material parameters to replicate average experimental data is likely to reflect the variance in knee geometry as much as it is to reflect the variance in material behaviour. This is especially true if measuring only contact behaviour or when there are uncertainties in the meniscus geometry or the knee alignment. The calibration of material parameters would benefit from local displacement information, where possible obtained close to the transition between the meniscus tissue and the ligamentous tissue of its horns, as well as in the area where the meniscus is expected to move the most, which depends on the type of loads. Displacements close to the horns can be used to verify or calibrate computational assumptions related to the meniscus anchorage to the tibia whereas maximum displacement values can be related to the material parameters.

The findings also provide an insight into the sources of variability in contact mechanics of the tibiofemoral joint. They confirm that meniscus shape is a major contributing factor in contact mechanics even when the kinematics are highly constrained (Meakin et al. 2013 and Haut Donahue et al. 2004) and therefore that alterations to meniscus shape following trauma or intervention are likely to cause changes to the contact mechanics of the knee and cartilage damage (Makris et al. 2011; Zhang et al. 2014; Ghouri et al. 2022). They also suggest that differences in meniscus health which would cause only a change of meniscus elasticity may lead to measurable changes in the force distribution between condyles and in the local displacement of the meniscus but generally cause little changes in contact area. Finally, they suggest that disruptions to the meniscal attachments to the tibia are unlikely to be translated in contact mechanics changes when the joint is constrained.

## Conclusion

Through a systematic sensitivity analysis, this work demonstrated the importance of the meniscus shape and joint alignment for modelling knee contact mechanics and meniscus kinematics, over that of the material parameters used for the meniscus or the assumptions made for root attachments, the latter having a slight effect on meniscus kinematics.

As such, when developing specimen-specific models for predicting global knee behaviour, the knee geometry needs to be specific to the specimen while generic material properties may be sufficient. It also means that, when calibrating computational model material parameters on experimental data, not only is it better to use data obtained for the specific knee, but also local displacement information is necessary.

## Data Availability

All new data associated with this study are openly available from the University of Leeds data repository (Yao and Mengoni 2023); the baseline models it utilises, and associated experimental data, are openly available (Cooper et al. 2023). More data related to the wider programme of work is available at Conaghan et al. (2020).
